# Knowledge, Attitudes, and practices regarding the anti-inflammatory diet among the general population

**DOI:** 10.3389/fnut.2026.1890302

**Published:** 2026-08-25

**Authors:** Pengyan Wu, Yi Liang, Jianlan Pan, Yu Feng, Yuxi Li, Wenyan Yang, Xiaoxiao Qian

**Affiliations:** Clinical Nutrition Department, Affiliated Hospital of Guizhou Medical University, Guiyang, China

**Keywords:** anti-Inflammatory agents, cross-sectional studies, diet, health knowledge, attitudes, practice, nutrition assessment

## Abstract

**Objective:**

This study aimed to investigate knowledge, attitudes, and practices (KAP) regarding the anti-inflammatory diet among the general population.

**Methods:**

A multicenter cross-sectional survey was conducted among adults in Guizhou, China, from February to April 2026. Demographic characteristics and KAP dimensions were assessed using a validated questionnaire. KAP levels were categorized according to Bloom’s criteria as poor (<60% of the total score), moderate (60–79%), or good (≥80%). Structural equation modeling (SEM) was used to explore the relationships among knowledge, attitude, and practice.

**Results:**

A total of 679 participants were included, predominantly aged 31–45 years. The mean knowledge, attitude, and practice scores were 10.36 ± 4.44 (possible range: 0–21), 31.38 ± 4.49 (possible range: 9–45), and 25.89 ± 4.94 (possible range: 8–40), respectively. Significant correlations were observed among the three dimensions (knowledge–attitude *r* = 0.274; knowledge–practice *r* = 0.451; attitude–practice *r* = 0.244; all *P* < 0.001). SEM indicated significant direct associations of knowledge with attitude (β = 0.482, *P* = 0.013) and practice (β = 0.486, *P* = 0.006), as well as an indirect association with practice through attitude (β = 0.185, *P* = 0.013).

**Conclusion:**

Participants demonstrated inadequate knowledge, positive attitudes, and moderate practices toward the anti-inflammatory diet. Higher knowledge was associated with more favorable attitudes and healthier dietary behaviors. Strengthening public access to accurate dietary information and implementing practical, low-cost educational strategies may enhance population-level adherence to anti-inflammatory dietary patterns.

## Introduction

Chronic low-grade inflammation has been increasingly recognized as a central mechanism underlying the development and progression of major noncommunicable diseases (1, 2). These inflammation-related chronic conditions collectively account for a substantial proportion of the global disease burden, contributing to more than 50% of all deaths worldwide, including ischemic heart disease, stroke, diabetes mellitus, chronic kidney disease, non-alcoholic fatty liver disease, and various autoimmune and neurodegenerative disorders (3). Given this context, dietary strategies designed to mitigate inflammation have gained increasing attention in disease prevention and health promotion (4). The anti-inflammatory diet is characterized by a high intake of fruits, vegetables, whole grains, legumes, nuts, and omega-3-rich fish, together with reduced consumption of processed foods, refined carbohydrates, and saturated fats (5). Accumulating evidence suggests that adherence to such a dietary pattern may beneficially modulate gut microbiota composition and reduce circulating inflammatory biomarkers, including C-reactive protein, interleukin-6, and tumor necrosis factor-α (6, 7).

Although growing evidence supports the potential health benefits of these dietary approaches, their population-level effectiveness also depends on public understanding, acceptance, and adoption. However, public awareness, understanding, and long-term adherence remain suboptimal. For example, a recent survey among 326 Polish parents found that 50.8% rated their knowledge of the anti-inflammatory diet as unsatisfactory, 62.8% did not know which foods should be included in such a diet, and 76.6% were unable to identify natural products with anti-inflammatory activity (8). Similarly, in a non-Mediterranean multi-ethnic adult sample in Australia, less than half of participants were aware of Mediterranean diet guidelines, food choices, and health benefits, and only 20% showed high adherence to this dietary pattern (9). These findings collectively underscore a persistent gap in nutrition education and public health communication, highlighting the urgent need for systematic investigation.

The Knowledge–Attitude–Practice (KAP) model offers a well-established theoretical framework for evaluating individuals’ health-related cognition and behaviors (10). It assumes that adequate knowledge shapes positive attitudes, which subsequently foster favorable health practices (11). KAP surveys have been widely applied to explore health behaviors in areas such as chronic disease prevention, nutrition education, and lifestyle modification (12). In the context of diet management, previous KAP studies have mainly focused on general dietary management among populations requiring specialized nutritional support, such as individuals with digestive system disorders (13, 14), or cancer (15), or other conditions requiring tailored dietary guidance. By contrast, anti-inflammatory diet-related KAP among the general population remains insufficiently investigated, particularly in Guizhou, China. This regional focus is particularly relevant because previous research involving Guizhou and other provinces in Southwest China reported generally low awareness of the Chinese Dietary Guidelines, with substantially lower awareness among rural than urban residents, highlighting regional and sociodemographic disparities in access to dietary knowledge. Evidence is especially limited regarding public understanding of the relationship between diet and chronic inflammation, willingness to adopt anti-inflammatory dietary patterns, and the associations among dietary knowledge, attitudes, and self-reported practices in this region.

Assessing these dimensions may help identify specific knowledge gaps and barriers to dietary adoption and inform targeted nutrition education strategies. Therefore, this study aimed to (1) assess knowledge, attitudes, and practices regarding the anti-inflammatory diet among adults in Guizhou Province; (2) identify demographic factors associated with KAP levels; and (3) explore the relationships among knowledge, attitudes, and practices using structural equation modeling. The present study may provide preliminary evidence on public KAP status and the pathways linking these dimensions.

## Methods

### Study design and participants

This cross-sectional study was conducted from February to April 2026. The questionnaire was administered across multiple settings in Guizhou, China, including the Affiliated Hospital of Guizhou Medical University, nearby universities, and adjacent factories, to capture respondents from the general adult population aged 18 years or older. Ethical approval was obtained from the Medical Ethics Committee of Affiliated Hospital of Guizhou Medical University, and informed consent was secured from all participants prior to data collection. Individuals with severe comorbid conditions such as advanced malignancies, significant cardiopulmonary disorders, or cognitive impairment were excluded to ensure the accuracy and reliability of questionnaire responses.

### Questionnaire

The questionnaire was developed with reference to established literature (16) and relevant guidelines (17). After the initial draft was prepared, it was reviewed by two specialists from our Department of Clinical Nutrition, who had 5 and 30 years of professional experience, respectively. As clinical nutrition specialists in our department, they assisted in the systematic refinement of the questionnaire items. They evaluated the relevance, clarity, and comprehensibility of each item and provided suggestions for improvement. Based on their feedback, repetitive and ambiguous descriptions were removed, the questionnaire was streamlined, and overly technical expressions were revised into more understandable language for the general population. A pilot survey involving 32 participants was subsequently conducted, followed by assessments of reliability and validity. The overall Cronbach’s α coefficient was 0.847, with corresponding values of 0.874, 0.860, and 0.787 for the knowledge, attitude, and practice sections, respectively, demonstrating satisfactory internal consistency across the instrument and its subscales. Construct validity was further evaluated using confirmatory factor analysis (CFA), which examined whether the proposed three-dimensional structure of knowledge, attitude, and practice was supported by the questionnaire data. Model fit was assessed using CMIN/DF, the root mean square error of approximation (RMSEA), the incremental fit index (IFI), the Tucker–Lewis index (TLI), and the comparative fit index (CFI). CMIN/DF values below 3 and RMSEA values below 0.08 were considered indicative of acceptable absolute fit, whereas IFI, TLI, and CFI values approaching or exceeding 0.90 were considered indicative of acceptable incremental fit (Supplementary Table 1). Factor loadings were also examined to assess the contribution of individual items to their corresponding domains (Supplementary Table 2).

The final questionnaire, administered in Chinese, comprised four components: demographic characteristics (including age, sex, educational attainment, marital status, monthly income, height, weight, chronic disease history, duration of physical activity, sleep condition, and information source), a knowledge section, an attitude section, and a practice section. Age was categorized into 18–30, 31–45, and >45 years to reflect young, middle-aged, and older adult groups while retaining adequate sample sizes in each category. Monthly income was self-reported by participants and categorized into four groups (<2000 RMB, 2000–5000 RMB, 5000–10000 RMB, and >10000 RMB) based on income classification approaches commonly used in previous KAP studies among general populations in China (18). The knowledge section included six single-choice items and nine true/false items. Single-choice responses “Very familiar,” “Have heard of it,” and “Not clear” were scored as 2, 1, and 0 points, respectively, while true/false items awarded 1 point for correct and 0 points for incorrect responses, yielding a total possible score of 0–21. The attitude section consisted of nine items rated on a five-point Likert scale ranging from 5 (strongly agree) to 1 (strongly disagree), with total scores ranging from 9 to 45. The practice section contained eight items scored from 1 (never) to 5 (always), generating a total score of 8–40. In addition, the practice domain included two supplementary questions – one assessing difficulties encountered when adhering to an anti-inflammatory diet and another identifying sources from which participants obtained dietary information. These items were analyzed descriptively and were not incorporated into the scoring system. In accordance with Bloom’s cut-off criteria (19, 20), which is widely adopted in KAP studies, each KAP dimension was classified as poor (<60% of the total score), moderate (60–79%), or good (≥80%).

### Questionnaire distribution and quality control

This study employed a convenience sampling approach. Participants were recruited from one hospital, nearby universities, and two enterprises in Guizhou, China. At the hospital, questionnaires were distributed face-to-face in hospital wards and outpatient clinics. At the universities and enterprises, eligible participants were invited on site and completed either paper-based questionnaires or the electronic questionnaire. The online questionnaire was administered through the Wenjuanxing platform and was disseminated mainly through WeChat groups, institutional work groups, and personal social networks. Before participation, all respondents received information regarding the study objectives, questionnaire content, and completion procedures. Four research assistants who received standardized training were responsible for questionnaire distribution and collection and provided clarification when necessary to ensure that participants understood the questionnaire items.

To safeguard data integrity and enhance the reliability of the findings, several screening procedures were implemented to identify and exclude invalid or biased responses. Duplicate submissions were restricted during data collection, and potential duplicate or invalid responses were further reviewed manually. Questionnaires completed in under 90 s were removed due to the high likelihood of insufficient comprehension or inattentive responding. Surveys containing implausible or erroneous entries such as unrealistic age values were also excluded to reduce systematic errors and limit the influence of extreme outliers. In addition, questionnaires displaying patterned or repetitive response behavior (e.g., selecting the same option for all items) were excluded because such patterns suggested a lack of genuine engagement with the survey.

### Sample size

A single-population proportion formula, n = [(Zα/2)^2^ × P (1–P)]/d^2^, was applied to estimate the required sample size. Because no prior KAP studies on anti-inflammatory diets have been conducted among the Chinese general population, a conservative prevalence estimate of 50% was adopted to represent baseline awareness. Using a 95% confidence level and a 5% margin of error, the minimum calculated sample size was 384 participants. The theoretical sample size was 480 which includes an extra 20% to allow for invalid cases. The final sample of 679 participants exceeded both the minimum calculated sample size and the target sample size.

### Statistical analysis

Statistical analyses were conducted using SPSS version 26.0 (IBM, Armonk, NY, USA), and structural equation modeling was performed with AMOS version 24.0 (IBM, Armonk, NY, USA). The continuous variables were described as means ± standard deviations (SD). The normal distribution of continuous data was checked using the Kolmogorov–Smirnov test. The continuous variables conforming to the normal distribution analyzed using Student’s *t*-test or ANOVA. Those with a skewed distribution were analyzed using the Wilcoxon–Mann–Whitney *U*-test or the Kruskal–Wallis analysis of variance. The categorical variables were described as *n* (%). Correlations among knowledge, attitude, and practice scores were examined using Spearman’s rank correlation coefficient. Structural equation modeling (SEM) was applied based on the hypothesized KAP framework to examine the direct paths from knowledge to attitude and practice, the path from attitude to practice, and the potential mediating role of attitude. Specifically, the model hypothesized that knowledge would be associated with attitude and practice, and that attitude would mediate the relationship between knowledge and practice. SEM was used to explore the relationships among these dimensions rather than to establish causal relationships. Model fit was assessed using the RMSEA, SRMR, TLI, and CFI. The interpretation of model fit considered commonly recommended criteria, with RMSEA and SRMR values below 0.08 indicating acceptable fit, and TLI and CFI values approaching or exceeding 0.90 generally considered indicative of acceptable fit. Because this study used a convenience sample and an exploratory framework, model fit indices were interpreted cautiously rather than as evidence of definitive model confirmation. A two-sided P-value < 0.05 was deemed statistically significant.

## Results

Initially, this study collected a total of 689 questionnaires. The following samples were excluded: 8 questionnaires with a response time of less than 90 s, 1 case aged below 18 years, and 1 case with an invalid entry (“485”). Ultimately, 679 valid questionnaires remained, with an effective rate of 98.55%.

### Demographic information on participants

This study included 679 participants from the general population with a relatively balanced gender distribution (49.2% male, 50.8% female). The cohort predominantly consisted of young and middle-aged adults, with the largest proportion aged 31–45 years (53.9%). Most participants were urban residents (62.3%) and married (71.3%). The population was generally healthy, with 85.0% reporting no chronic conditions, though hypertension (10.6%) and hyperlipidemia (6.9%) were the most common comorbidities among those with health issues. Their mean knowledge, attitude, and practice scores were 10.36 ± 4.44, 31.38 ± 4.49, and 25.89 ± 4.94, respectively. Knowledge scores differed significantly by education level (*P* < 0.001), marital status (*P* = 0.027), and monthly income (*P* < 0.001). Attitude scores varied significantly with gender (*P* < 0.001), residence (*P* < 0.001), education level (*P* < 0.001), monthly income (*P* < 0.001), marital status (*P* = 0.019), BMI (*P* = 0.006), and sleep condition (*P* < 0.001). Practice scores showed significant associations with age (*P* = 0.012), residence (*P* < 0.001), education level (*P* < 0.001), monthly income (*P* < 0.001), and diabetes status (*P* = 0.024) (Table 1).

**TABLE 1:** Demographic characteristics and KAP scores.

Variables	*N* (%)	Knowledge		Attitude		Practice	
		Mean ± SD	*P*	Mean ± SD	*P*	Mean ± SD	*P*
*N* = 679		10.36 ± 4.44		31.38 ± 4.49		25.89 ± 4.94	
Gender			0.742		<0.001		0.228
Male	334 (49.19)	10.31 ± 4.29		30.5 ± 4.37		25.62 ± 4.9	
Female	345 (50.81)	10.41 ± 4.58		32.22 ± 4.44		26.16 ± 4.97	
Age (year)			0.359		0.243		0.012
18–30	230 (33.87)	10.06 ± 4.34		31.72 ± 4.53		25.36 ± 4.46	
31–45	386 (53.90)	10.6 ± 4.13		31.17 ± 4.61		25.84 ± 4.99	
>45	83 (12.22)	10.12 ± 5.82		31.35 ± 3.73		27.6 ± 5.59	
Residence			0.085		<0.001		<0.001
Rural	256 (37.7)	10.07 ± 3.96		30.53 ± 4.39		24.84 ± 4.76	
Urban	423 (62.3)	10.53 ± 4.7		31.89 ± 4.47		26.53 ± 4.94	
Education			<0.001		<0.001		< 0.001
High school / Technical secondary school or below	85 (12.52)	8.19 ± 4.25		29.25 ± 3.92		24.52 ± 4.83	
Associate degree	117 (17.23)	8.56 ± 5.08		32.91 ± 5.39		23.93 ± 5.1	
Bachelor’s degree	402 (59.2)	10.71 ± 3.67		31.06 ± 4.26		25.91 ± 4.32	
Master’s degree or above	75 (11.05)	13.75 ± 4.88		33.09 ± 3.25		30.44 ± 5.08	
Marital status			0.027		0.019		0.060
Unmarried	195 (28.72)	9.73 ± 4.7		32.04 ± 4.54		25.26 ± 5.03	
Married	484 (71.28)	10.61 ± 4.31		31.11 ± 4.44		26.15 ± 4.88	
Monthly income per capita (RMB)			<0.001		<0.001		<0.001
<2,000	91 (13.4)	7.87 ± 4.99		31.96 ± 5.36		24.26 ± 4.46	
2,000–5,000	189 (27.84)	9.78 ± 4.1		31.37 ± 4.91		24.98 ± 4.94	
5,000–10,000	328 (48.31)	10.79 ± 4.09		30.76 ± 3.85		26.28 ± 4.6	
>10,000	71 (10.46)	13.07 ± 4.25		33.49 ± 4.14		28.63 ± 5.69	
BMI (m/kg^2^)			0.343		0.006		0.349
<18.5	81 (11.93)	10.75 ± 3.81		30.47 ± 4.13		26.19 ± 4.35	
18.5–24.9	467 (68.78)	10.42 ± 4.47		31.28 ± 4.42		25.95 ± 4.81	
25–27.9	90 (13.25)	10.37 ± 4.26		31.52 ± 4.38		25.88 ± 5.85	
>27.9	41 (6.04)	8.9 ± 5.34		33.88 ± 5.38		24.68 ± 5.33	
Hypertension			0.172		0.286		0.585
Yes	72 (10.6)	10.78 ± 4.91		31.69 ± 3.79		26.11 ± 4.85	
No	607 (89.4)	10.31 ± 4.38		31.34 ± 4.56		25.87 ± 4.95	
Hyperlipidemia			0.127		0.130		0.078
Yes	47 (6.92)	11.04 ± 4.78		31.96 ± 4.14		26.89 ± 5.21	
No	632 (93.08)	10.31 ± 4.41		31.33 ± 4.51		25.82 ± 4.91	
Coronary heart disease			0.105		0.557		0.385
Yes	21 (3.09)	12.05 ± 4.72		30.57 ± 2.36		26.9 ± 5.43	
No	658 (96.91)	10.3 ± 4.42		31.4 ± 4.54		25.86 ± 4.92	
*N* = 679		10.36 ± 4.44		31.38 ± 4.49		25.89 ± 4.94	0.228
Gender			0.742		<0.001		
Diabetes			0.147		0.991		0.024
Yes	36 (5.3)	11.25 ± 4.44		31.14 ± 4.31		27.53 ± 4.91	
No	643 (94.7)	10.31 ± 4.44		31.39 ± 4.5		25.8 ± 4.93	
Time spent on physical activity each day			0.285		0.205		0.426
0 h	108 (15.91)	10.06 ± 4.06		30.44 ± 3.49		25.25 ± 4.16	
0–1 h	328 (48.31)	10.66 ± 4.27		31.57 ± 4.41		26.11 ± 4.93	
1–2 h	158 (23.27)	10.28 ± 4.82		31.27 ± 4.29		26.03 ± 5.24	
More than 2 h	85 (12.52)	9.71 ± 4.77		32.02 ± 5.92		25.6 ± 5.27	
Sleep condition			0.962		<0.001		0.673
Good	560 (82.47)	10.44 ± 4.32		31.07 ± 4.35		25.94 ± 4.83	
Poor, frequent insomnia or awakenings	119 (17.53)	9.99 ± 4.98		32.82 ± 4.85		25.69 ± 5.43	

### Distribution of responses to knowledge, attitude, and practice

Overall, participants demonstrated limited familiarity with the concept and specific characteristics of the anti-inflammatory diet. Although some participants recognized the relationship between diet and inflammation and identified several anti-inflammatory foods, detailed knowledge regarding the definition, dietary composition, and recommended proportions of the anti-inflammatory diet remained insufficient (Table 2).

**TABLE 2:** Distribution of knowledge dimension responses.

Knowledge items, n (%)	Very familiar	Heard of it	Not sure
1. Various components in the diet can influence the body’s inflammatory processes, and inflammation levels can be regulated by changing dietary patterns.	60 (8.84)	379 (55.82)	240 (35.35)
2. The anti-inflammatory diet refers to a dietary pattern aimed at reducing chronic inflammation, emphasizing long-term and balanced intake of foods that help lower inflammation in the body.	45 (6.63)	361 (53.17)	273 (40.21)
3. Chronic inflammation is associated with various diseases such as heart disease, diabetes, arthritis, and certain cancers.	118 (17.38)	419 (61.71)	142 (20.91)
5. Whole-grain foods are rich in fiber, vitamins, and minerals, which help reduce inflammation levels in the body.	82 (12.08)	383 (56.41)	214 (31.52)
6. In an ideal anti-inflammatory diet, vegetables and fruits should account for two-thirds of the total food intake by weight.	62 (9.13)	272 (40.06)	345 (50.81)
7. Cooking methods in an anti-inflammatory diet should be healthy, mainly focusing on braising, stir-frying, steaming, and boiling, while minimizing frying, deep-frying, and grilling.	178 (26.22)	392 (57.73)	109 (16.05)
4. Are the following foods considered anti-inflammatory?	True	False	Not sure
4.1 Whole grains (e.g., coarse grains, whole wheat bread)	470 (69.22)	59 (8.69)	150 (22.09)
4.2 Deep-sea fish	424 (62.44)	82 (12.08)	173 (25.48)
4.3 Red meat (e.g., pork, beef)	215 (31.66)	264 (38.88)	200 (29.46)
4.4 Legumes	418 (61.56)	89 (13.11)	172 (25.33)
4.5 Green leafy vegetables	506 (74.52)	66 (9.72)	107 (15.76)
4.6 Nuts	434 (63.92)	89 (13.11)	156 (22.97)
4.7 Fried foods	168 (24.74)	379 (55.82)	132 (19.44)
4.8 Tea	393 (57.88)	119 (17.53)	167 (24.59)
4.9 Sugar-sweetened beverages	89 (13.11)	449 (66.13)	141 (20.77)

Participants generally expressed positive attitudes toward the anti-inflammatory diet, including recognition of its potential health benefits and willingness to acquire related knowledge. However, concerns regarding dietary complexity, difficulty in long-term adherence, and uncertainty about the magnitude of its benefits were also observed (Table 3).

**TABLE 3:** Distribution of attitude dimension responses.

Attitude items, *n* (%)	Strongly agree	Agree	Neutral	Disagree	Strongly disagree
1. I believe that an anti-inflammatory diet is more beneficial to health than a traditional diet.	133 (19.59)	301 (44.33)	162 (23.86)	79 (11.63)	4 (0.59)
2. I believe that an anti-inflammatory diet can effectively prevent certain diseases.	121 (17.82)	316 (46.54)	166 (24.45)	67 (9.87)	9 (1.33)
3. I am willing to change my eating habits to adopt an anti-inflammatory diet.	101 (14.87)	286 (42.12)	180 (26.51)	98 (14.43)	14 (2.06)
4. I believe that adjusting dietary habits can serve as an adjunct to medication therapy.	119 (17.53)	300 (44.18)	172 (25.33)	76 (11.19)	12 (1.77)
5. Since the anti-inflammatory diet requires control over various types of food intake, I find this dietary change difficult for me.	55 (8.1)	253 (37.26)	212 (31.22)	137 (20.18)	22 (3.24)
6. I believe that the anti-inflammatory diet is demanding and difficult to maintain over the long term.	74 (10.9)	258 (38)	201 (29.6)	129 (19)	17 (2.5)
7. I think the role of diet in controlling inflammation has been exaggerated.	34 (5.01)	158 (23.27)	292 (43)	173 (25.48)	22 (3.24)
8. I am interested in trying new anti-inflammatory recipes.	87 (12.81)	271 (39.91)	229 (33.73)	76 (11.19)	16 (2.36)
9. I am willing to learn more about anti-inflammatory diet–related knowledge and information.	109 (16.05)	306 (45.07)	201 (29.6)	49 (7.22)	14 (2.06)

Compared with their relatively positive attitudes, participants reported less favorable dietary practices. The main challenges included insufficient attention to anti-inflammatory properties during food selection, inconsistent consumption of recommended food groups, and difficulties avoiding processed or unhealthy foods. Economic cost, access to reliable information, dietary habit changes, time constraints, and food availability were frequently reported barriers to adherence (Table 4).

**TABLE 4:** Distribution of practice dimension responses.

Practice items, *n* (%)	Always	Often	Sometimes	Rarely	Never
1. When purchasing food, I pay attention to its anti-inflammatory properties.	46 (6.77)	133 (19.59)	240 (35.35)	160 (23.56)	100 (14.73)
2. I follow the dietary advice provided by a nutritionist or physician.	92 (13.55)	179 (26.36)	274 (40.35)	104 (15.32)	30 (4.42)
3. In my daily diet, I am able to:					
3.1 Regularly consume foods rich in Omega-3 fatty acids (e.g., deep-sea fish, nuts)	98 (14.43)	124 (18.26)	232 (34.17)	178 (26.22)	47 (6.92)
3.2 Eat whole-grain foods regularly	106 (15.61)	177 (26.07)	227 (33.43)	120 (17.67)	49 (7.22)
3.3 Avoid consuming processed foods and high-sugar foods as much as possible	103 (15.17)	175 (25.77)	237 (34.9)	138 (20.32)	26 (3.83)
3.4 Frequently eat fresh fruits and vegetables	148 (21.8)	250 (36.82)	181 (26.66)	67 (9.87)	33 (4.86)
3.5 Avoid long-term excessive or heavy alcohol consumption	183 (26.95)	159 (23.42)	161 (23.71)	103 (15.17)	73 (10.75)
3.6 Minimize the use of frying, deep-frying, and grilling when cooking	97 (14.29)	159 (23.42)	251 (36.97)	113 (16.64)	59 (8.69)

### Correlation analysis

Correlation analysis indicated significant positive correlations between knowledge and attitude (*r* = 0.274, *P* < 0.001), as well as practice (*r* = 0.451, *P* < 0.001). Meanwhile, there was also correlation between attitude and practice (*r* = 0.244, *P* < 0.001) (Supplementary Table 3).

### SEM analysis

The model showed adequate absolute fit based on CMIN/DF (2.840) and RMSEA (0.052), but the incremental fit indices remained below commonly recommended thresholds, indicating suboptimal overall model fit (Supplementary Table 4). The structural path estimates showed significant positive associations between knowledge and attitude (standardized estimate = 0.482, *P* < 0.001), knowledge and practice (standardized estimate = 0.486, *P* < 0.001), and attitude and practice (standardized estimate = 0.384, *P* < 0.001) (Supplementary Table 5). The bootstrap analysis further indicated direct associations of knowledge with attitude (β = 0.482, *P* = 0.013) and practice (β = 0.486, *P* = 0.006).

**FIGURE 1:**
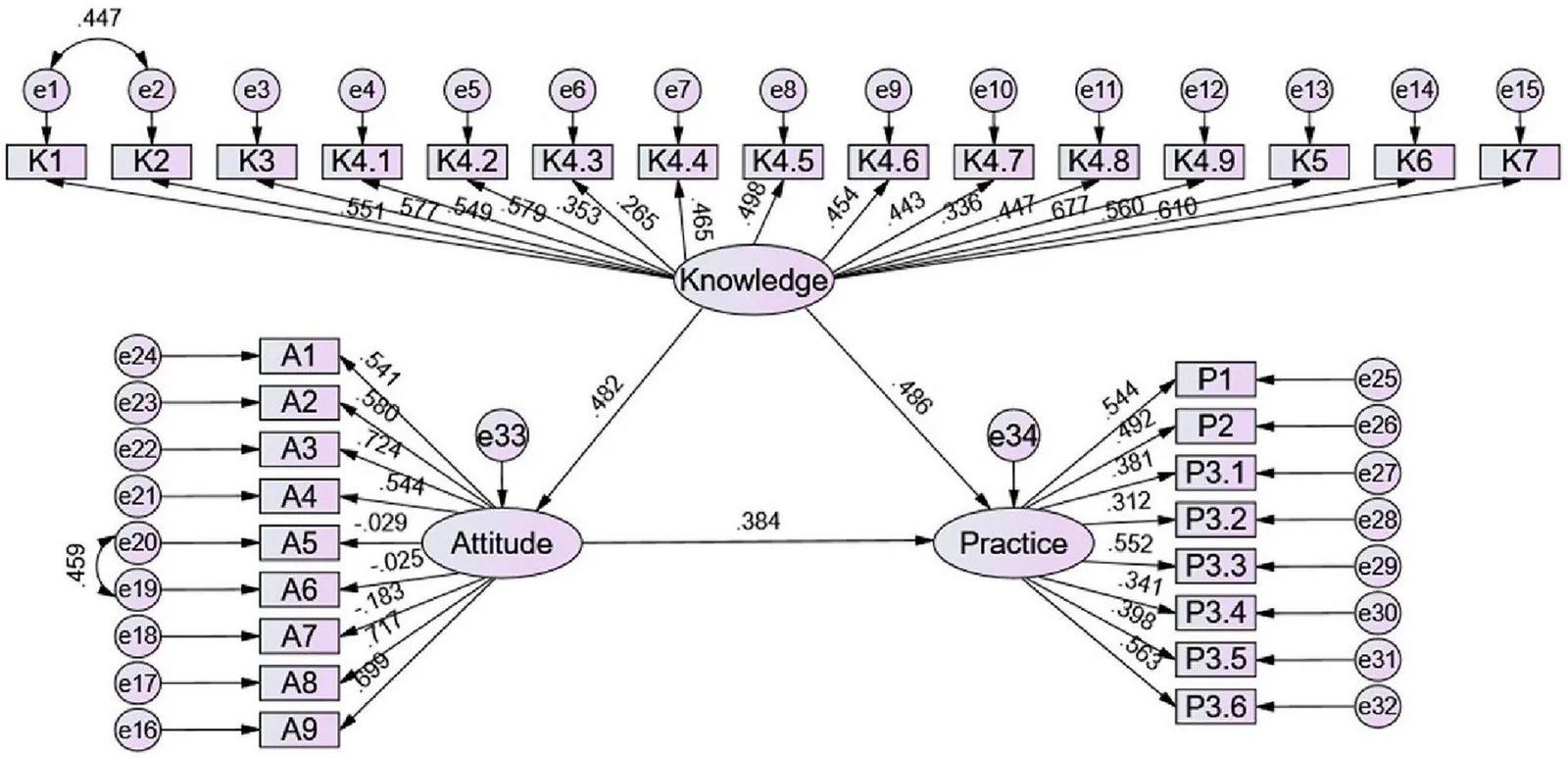
Structural Equation Model (SEM) depicting the relationships among the latent variables: knowledge, attitude, and practice. Ellipses represent latent variables (unobserved constructs), including overall knowledge, attitude, and practice. Rectangles represent observed variables (measured questionnaire items) that serve as indicators of the latent variables. Circles represent error terms or residuals associated with the observed variables. Single-headed arrows indicate hypothesized directional relationships (pathways) between latent variables and from latent variables to observed variables, with standardized path coefficients labeled on the arrows.

Meanwhile, attitude was directly associated with practice (β = 0.384, *P* = 0.008). Furthermore, an indirect association between knowledge and practice through attitude was observed (β = 0.185, *P* = 0.015) (Figure 1 and Table 5).

**TABLE 5:** SEM direct and indirect effects.

Model paths	Standardized Total effects		Standardized direct effects		Standardized indirect effects	
	β (95%CI)	*P*	β (95%CI)	*P*	β (95%CI)	*P*
Knowledge → Attitude	0.482 (0.371–0.573)	0.013	0.482 (0.371–0.573)	0.013		
Knowledge → Practice	0.671 (0.580–0.749)	0.009	0.486 (0.381–0.612)	0.006		
Attitude → Practice	0.384 (0.270–0.488)	0.008	0.384 (0.270–0.488)	0.008		
Knowledge → Practice					0.185 (0.113–0.255)	0.015

## Discussion

### Overall KAP status regarding the anti-inflammatory diet

Participants exhibited inadequate knowledge, positive attitudes, and suboptimal practices regarding the anti-inflammatory diet, and higher knowledge levels were associated with more favorable attitudes and healthier dietary behaviors. The present study should be regarded as a preliminary exploration of public KAP regarding the anti-inflammatory diet and the relationships among these dimensions, rather than a comprehensive assessment of actual dietary intake or clinical anti-inflammatory effects. This pattern is consistent with previous dietary KAP studies showing that favorable knowledge and attitudes do not necessarily translate into sustained dietary behaviors (18, 21, 22). These findings suggest that practical and contextual barriers should also be considered when promoting anti-inflammatory dietary patterns.

### Relationships among knowledge, attitudes, and practices

In this study, knowledge was positively associated with both attitude and practice, and attitude was positively associated with practice. SEM further suggested potential direct and indirect associations between knowledge, attitude, and practice through attitude pathways. Given the cross-sectional design and convenience sampling approach, these findings should be interpreted as exploratory associations rather than causal relationships. These findings provide preliminary evidence on the relationships among anti-inflammatory diet-related knowledge, attitudes, and self-reported practices in the general population. They are also consistent with previous KAP studies suggesting that knowledge may be related to dietary behavior directly and through attitudinal pathways (23–25).

### Sociodemographic factors associated with KAP

The sociodemographic patterning of KAP scores also corresponds with trends reported elsewhere. Respondents with higher levels of education and income scored better on knowledge and practice, in line with studies from different regions of China and other middle-income settings where more educated and economically advantaged groups generally achieve healthier diet quality and more favorable KAP profiles (18). Research among older adults and people with multiple chronic conditions suggests that individuals with greater educational attainment are more likely to recognize diet–disease links, to seek professional advice and to enact complex dietary prescriptions over time (18). Gender, body weight status and sleep quality showed distinct associations with attitudes in this survey, mirroring work in which women and those with greater health consciousness often report more concern about diet, but also higher perceived burden and worry about long-term restriction (18, 22). Respondents with diabetes in this sample reported better practices, which echoes studies indicating that those already under medical follow-up for metabolic diseases may be more engaged with dietary advice, although this engagement can remain fragile without continued support (18, 24). Residence also contributed to variation in KAP scores, with urban participants showing better attitudes and practices than rural residents. These differences may reflect disparities in socioeconomic resources, access to healthcare and nutrition information, food availability, and opportunities to apply dietary guidance in everyday life. The better practice scores observed among participants with diabetes may also be related to more frequent contact with healthcare services and greater exposure to dietary guidance. These findings highlight the importance of considering local social and healthcare conditions when interpreting anti-inflammatory diet-related KAP in Guizhou Province.

### Knowledge gaps, attitudes, and barriers to dietary practice

Item-level analysis of knowledge responses highlights a specific gap: only a minority of respondents felt truly familiar with the concept of an anti-inflammatory diet, its focus on chronic low-grade inflammation and its emphasis on plant-rich patterns with generous portions of fruits and vegetables. In contrast, more respondents could correctly link chronic inflammation with major noncommunicable diseases and identify some anti-inflammatory food categories. Surveys of Mediterranean and healthy diet knowledge in community samples report similar discrepancies, where people more readily endorse the importance of diet for preventing cardiovascular and metabolic disease yet struggle to specify recommended food group proportions or to classify particular foods correctly (21, 22). Studies of nutrition knowledge in older adults also illustrate that understanding of general principles, such as “eating more vegetables,” tends to outpace familiarity with specific dietary models and their operational definitions (18, 22). These findings suggest that educational messages on anti-inflammatory nutrition may need to move beyond broad statements about “healthy eating” toward more concrete depictions of the dietary pattern, using simple visual tools and context-appropriate examples rather than technical terminology alone (18, 21, 22).

The attitude items depict a group that largely accepts the health value of the anti-inflammatory diet but perceives the approach as demanding, difficult to sustain and, for some, perhaps over-claimed in its benefits. Comparable surveys of Mediterranean diet attitudes among older adults show that many participants describe the diet as attractive in theory yet restrictive or complex when applied to their own routines, particularly when social eating, cultural preferences and family habits are considered (22). Cross-national work on Mediterranean diet adherence has identified perceived inconvenience, the effort required for meal planning and cooking, and a sense that dietary prescriptions compete with other life priorities as key drivers of ambivalence, even among individuals who intellectually endorse the benefits of the pattern (26). Clinical KAP research adds an additional layer by showing that patients facing multiple dietary restrictions often experience a degree of skepticism toward dietary claims, especially when they have encountered conflicting messages from various sources (24). The pattern of positive yet guarded attitudes in this study therefore resembles broader observations that people may accept diet–health links in general but maintain reservations about feasibility and the proportionality of dietary recommendations in the context of their everyday lives (22, 24, 26).

Dietary practices in this sample were less favorable than attitudes, with many respondents’ reporting inconsistent attention to anti-inflammatory properties when purchasing food, intermittent consumption of omega-3-rich and whole-grain foods, and limited efforts to avoid processed, sugary or heavily fried items. Financial constraints, difficulties in accessing reliable information, entrenched eating habits, time pressure and limited availability of specific foods emerged as prominent barriers. Investigations of healthy eating and Mediterranean diet adherence across various countries consistently highlight cost, convenience, taste preferences and food availability as obstacles to maintaining recommended patterns, particularly for nutritionally dense items such as fresh fish, nuts and high-quality produce (21, 26). Economic analyses in China indicate that full adherence to national dietary guidelines often requires a higher food budget, which can place recommended diets out of reach for low-income households despite strong health messaging (27). Studies of urban poor communities show that food environment constraints, including limited retail options and higher prices for perishable foods, reinforce energy-dense, nutrient-poor choices even among individuals who express a desire to eat more healthily (28). The present findings, with cost, limited time, and difficulty accessing reliable information emerging as major barriers, suggest that dietary practices may be shaped not only by individual knowledge and motivation but also by broader social and environmental conditions. These barriers highlight the importance of considering household economic resources, access to trustworthy nutrition information, and the local food environment when promoting anti-inflammatory dietary patterns (21, 26).

The role of information sources in this study also deserves attention. Many participants reported learning about the anti-inflammatory diet through healthcare providers, hospital-based education and new media platforms. KAP studies in China have shown that contact with community doctors, dietitians or structured health education sessions tends to correlate with higher nutrition knowledge scores and more favorable practices, particularly among middle-aged and older adults (18). At the same time, research on online health information has documented variability in the quality and accuracy of nutrition content circulated through social media and commercial platforms, which can create confusion or skepticism, especially when messages about specific “anti-inflammatory” foods are exaggerated or contradictory (21, 22). Effective intervention strategies in this domain therefore need to harness trusted healthcare channels while also engaging with digital media in a way that filters, standardizes and amplifies evidence-based content, rather than leaving individuals to navigate a crowded and sometimes misleading information environment (18, 21).

The findings suggest that nutrition interventions should prioritize individuals with lower educational attainment and income, as well as rural residents. Educational content should focus on the basic concept of the anti-inflammatory diet, identification of relevant food groups, recommended dietary composition, and practical food choices. Given the reported barriers related to cost, limited time, and difficulty accessing reliable information, community- and primary care-based programs should provide simple, affordable, and locally applicable dietary guidance, including practical meal planning and cooking strategies (18, 27–29).

This study has several limitations. First, the cross-sectional design precludes causal inference and does not allow the temporal relationships among knowledge, attitudes, and practices to be established. Therefore, the associations and pathways identified in this study should be interpreted as exploratory rather than causal. Second, although the final sample size exceeded the minimum required sample size, convenience sampling from a single province and recruitment through hospitals, universities, and enterprises may have introduced selection bias and limited population representativeness. In addition, recruitment-site and survey-mode identifiers were not recorded during data collection; therefore, we were unable to determine the exact number and proportion of participants recruited from each setting, calculate recruitment-specific response or loss rates, or compare differences between online and offline respondents. Consequently, the findings should be interpreted cautiously and should not be directly generalized to the overall Chinese population. Third, self-reported questionnaire data are subject to recall bias and social desirability bias, which could lead to overestimation or underestimation of actual dietary behaviors. In addition, actual dietary intake was not assessed using objective or detailed dietary assessment methods. Fourth, although the questionnaire demonstrated satisfactory internal consistency, the CFA showed acceptable absolute fit but suboptimal incremental fit, and several items had low or nonsignificant factor loadings. Therefore, the proposed questionnaire structure should be interpreted cautiously and requires further refinement and validation in more diverse populations and settings. Future studies should incorporate more comprehensive dietary assessment methods, such as detailed dietary intake evaluation or longitudinal dietary monitoring, to further clarify the relationship between anti-inflammatory dietary patterns, behavioral changes, and inflammation-related health outcomes.

Although the general population exhibits generally positive attitudes toward the anti-inflammatory diet, knowledge and actual dietary practices remain only moderate or inadeauqte, with knowledge showing both direct and indirect associations with behavior through attitudes. Integrating structured, evidence-based anti-inflammatory nutrition education and behavior-support strategies into routine primary care and chronic disease follow-up may support adherence to anti-inflammatory dietary patterns. However, the effects of these strategies on dietary adherence and inflammation-related health outcomes require confirmation in longitudinal and intervention studies.

## Data Availability

The original contributions presented in the study are included in the article/Supplementary material, further inquiries can be directed to the corresponding author.
