# Supplementary material for: Knowledge, Attitudes, and practices regarding the anti-inflammatory diet among the general population
Source: Front Nutr. 2026 Aug 25;13:1890302. doi: 10.3389/fnut.2026.1890302 (PMC13549841; doi:10.3389/fnut.2026.1890302)
Supplement: Supplementary file 1 [file Table_1.docx]

**Supplementary Table 1. Model fit indices in the confirmatory factor analysis**

| Indicators | Reference | Actual |
| --- | --- | --- |
| CMIN/DF | 1-3: Excellent, 3-5: Good | 2.840 |
| RMSEA | <0.08: Good | 0.052 |
| IFI | >0.8: Good | 0.830 |
| TLI | >0.8: Good | 0.815 |
| CFI | >0.8: Good | 0.828 |

**Supplementary Table 2. Standardized and unstandardized factor loadings in the confirmatory factor analysis**

|  |  |  | **Estimate** | **Standardized Estimate** | **S.E.** | **C.R.** | **P** |
| --- | --- | --- | --- | --- | --- | --- | --- |
| K1 | <--- | Knowledge | 1.000 | 0.551 |  |  |  |
| K2 | <--- | Knowledge | 1.024 | 0.577 | 0.066 | 15.408 | <0.001 |
| K3 | <--- | Knowledge | 1.010 | 0.549 | 0.092 | 11.008 | <0.001 |
| K4.1 | <--- | Knowledge | 0.796 | 0.579 | 0.070 | 11.418 | <0.001 |
| K4.2 | <--- | Knowledge | 0.510 | 0.353 | 0.065 | 7.816 | <0.001 |
| K4.3 | <--- | Knowledge | 0.385 | 0.265 | 0.063 | 6.072 | <0.001 |
| K4.4 | <--- | Knowledge | 0.674 | 0.465 | 0.069 | 9.759 | <0.001 |
| K4.5 | <--- | Knowledge | 0.646 | 0.498 | 0.063 | 10.269 | <0.001 |
| K4.6 | <--- | Knowledge | 0.650 | 0.454 | 0.068 | 9.586 | <0.001 |
| K4.7 | <--- | Knowledge | 0.656 | 0.443 | 0.070 | 9.408 | <0.001 |
| K4.8 | <--- | Knowledge | 0.494 | 0.336 | 0.066 | 7.489 | <0.001 |
| K4.9 | <--- | Knowledge | 0.630 | 0.447 | 0.067 | 9.459 | <0.001 |
| K5 | <--- | Knowledge | 1.272 | 0.677 | 0.101 | 12.599 | <0.001 |
| K6 | <--- | Knowledge | 1.089 | 0.560 | 0.097 | 11.168 | <0.001 |
| K7 | <--- | Knowledge | 1.166 | 0.610 | 0.099 | 11.814 | <0.001 |
| A9 | <--- | Attitude | 1.000 | 0.699 |  |  |  |
| A8 | <--- | Attitude | 1.062 | 0.717 | 0.067 | 15.927 | <0.001 |
| A7 | <--- | Attitude | -0.262 | -0.183 | 0.061 | -4.328 | <0.001 |
| A6 | <--- | Attitude | -0.040 | -0.025 | 0.066 | -0.603 | 0.546 |
| A5 | <--- | Attitude | -0.045 | -0.029 | 0.065 | -0.685 | 0.494 |
| A4 | <--- | Attitude | 0.822 | 0.544 | 0.066 | 12.474 | <0.001 |
| A3 | <--- | Attitude | 1.124 | 0.724 | 0.070 | 16.045 | <0.001 |
| A2 | <--- | Attitude | 0.846 | 0.580 | 0.064 | 13.247 | <0.001 |
| A1 | <--- | Attitude | 0.798 | 0.541 | 0.064 | 12.410 | <0.001 |
| P1 | <--- | Practice | 1.000 | 0.544 |  |  |  |
| P2 | <--- | Practice | 0.828 | 0.492 | 0.088 | 9.367 | <0.001 |
| P3.1 | <--- | Practice | 0.713 | 0.381 | 0.092 | 7.748 | <0.001 |
| P3.2 | <--- | Practice | 0.582 | 0.312 | 0.089 | 6.565 | <0.001 |
| P3.3 | <--- | Practice | 0.969 | 0.552 | 0.096 | 10.100 | <0.001 |
| P3.4 | <--- | Practice | 0.605 | 0.341 | 0.085 | 7.074 | <0.001 |
| P3.5 | <--- | Practice | 0.860 | 0.398 | 0.107 | 8.012 | <0.001 |
| P3.6 | <--- | Practice | 1.050 | 0.563 | 0.103 | 10.233 | <0.001 |

**Supplementary table 3. Correlation analysis**

|  | Knowledge | Attitude | Practice |
| --- | --- | --- | --- |
| Knowledge | 1 |  |  |
| Attitude | 0.274 (P<0.001) | 1 |  |
| Practice | 0.451 (P<0.001) | 0.244 (P<0.001) | 1 |

**Supplementary table 4. SEM fit indicators**

| Indicators | Reference | Actual |
| --- | --- | --- |
| CMIN/DF | 1-3: Excellent, 3-5: Good | 2.840 |
| RMSEA | <0.08: Good | 0.052 |
| IFI | >0.8: Good | 0.830 |
| TLI | >0.8: Good | 0.815 |
| CFI | >0.8: Good | 0.828 |

**Supplementary Table 5. Structural path estimates and factor loadings**

|  |  |  | **Estimate** | **Standardized Estimate** | **S.E.** | **C.R.** | **P** |
| --- | --- | --- | --- | --- | --- | --- | --- |
| Attitude | <--- | Knowledge | 0.906 | 0.482 | 0.104 | 8.672 | <0.001 |
| Practice | <--- | Attitude | 0.371 | 0.384 | 0.056 | 6.646 | <0.001 |
| Practice | <--- | Knowledge | 0.881 | 0.486 | 0.119 | 7.414 | <0.001 |
| K1 | <--- | Knowledge | 1.000 | 0.551 |  |  |  |
| K2 | <--- | Knowledge | 1.024 | 0.577 | 0.066 | 15.408 | <0.001 |
| K3 | <--- | Knowledge | 1.010 | 0.549 | 0.092 | 11.008 | <0.001 |
| K4.1 | <--- | Knowledge | 0.796 | 0.579 | 0.070 | 11.418 | <0.001 |
| K4.2 | <--- | Knowledge | 0.510 | 0.353 | 0.065 | 7.816 | <0.001 |
| K4.3 | <--- | Knowledge | 0.385 | 0.265 | 0.063 | 6.072 | <0.001 |
| K4.4 | <--- | Knowledge | 0.674 | 0.465 | 0.069 | 9.759 | <0.001 |
| K4.5 | <--- | Knowledge | 0.646 | 0.498 | 0.063 | 10.269 | <0.001 |
| K4.6 | <--- | Knowledge | 0.650 | 0.454 | 0.068 | 9.586 | <0.001 |
| K4.7 | <--- | Knowledge | 0.656 | 0.443 | 0.070 | 9.408 | <0.001 |
| K4.8 | <--- | Knowledge | 0.494 | 0.336 | 0.066 | 7.489 | <0.001 |
| K4.9 | <--- | Knowledge | 0.630 | 0.447 | 0.067 | 9.459 | <0.001 |
| K5 | <--- | Knowledge | 1.272 | 0.677 | 0.101 | 12.599 | <0.001 |
| K6 | <--- | Knowledge | 1.089 | 0.560 | 0.097 | 11.168 | <0.001 |
| K7 | <--- | Knowledge | 1.166 | 0.610 | 0.099 | 11.814 | <0.001 |
| A9 | <--- | Attitude | 1.000 | 0.699 |  |  |  |
| A8 | <--- | Attitude | 1.062 | 0.717 | 0.067 | 15.927 | <0.001 |
| A7 | <--- | Attitude | -0.262 | -0.183 | 0.061 | -4.328 | <0.001 |
| A6 | <--- | Attitude | -0.040 | -0.025 | 0.066 | -0.603 | 0.546 |
| A5 | <--- | Attitude | -0.045 | -0.029 | 0.065 | -0.685 | 0.494 |
| A4 | <--- | Attitude | 0.822 | 0.544 | 0.066 | 12.474 | <0.001 |
| A3 | <--- | Attitude | 1.124 | 0.724 | 0.070 | 16.045 | <0.001 |
| A2 | <--- | Attitude | 0.846 | 0.580 | 0.064 | 13.247 | <0.001 |
| A1 | <--- | Attitude | 0.798 | 0.541 | 0.064 | 12.410 | <0.001 |
| P1 | <--- | Practice | 1.000 | 0.544 |  |  |  |
| P2 | <--- | Practice | 0.828 | 0.492 | 0.088 | 9.367 | <0.001 |
| P3.1 | <--- | Practice | 0.713 | 0.381 | 0.092 | 7.748 | <0.001 |
| P3.2 | <--- | Practice | 0.582 | 0.312 | 0.089 | 6.565 | <0.001 |
| P3.3 | <--- | Practice | 0.969 | 0.552 | 0.096 | 10.100 | <0.001 |
| P3.4 | <--- | Practice | 0.605 | 0.341 | 0.085 | 7.074 | <0.001 |
| P3.5 | <--- | Practice | 0.860 | 0.398 | 0.107 | 8.012 | <0.001 |
| P3.6 | <--- | Practice | 1.050 | 0.563 | 0.103 | 10.233 | <0.001 |
